# Giving radiologists a voice: a review of podcasts in radiology

**DOI:** 10.1186/s13244-020-0842-3

**Published:** 2020-03-04

**Authors:** Christopher G. D. Clarke, Uzoma Nnajiuba, Jamie Howie, Muhammad Khan, Daniel Pinto dos Santos, Erik Ranschaert

**Affiliations:** 1 Department of Radiology, Nottingham University Hospitals NHS Trust, Derby Road, Nottingham, NG7 2UH UK; 2 Department of Radiology, Aintree University Hospital NHS Foundation Trust, Liverpool, L9 7AL UK; 3 Department of Radiology, Faculty of Medicine and University Hospital, University of Cologne, Kerpener Str. 62, 50937 Cologne, Germany; 4 Department of Radiology, Elisabeth-TweeSteden Ziekenhuis, Hilvarenbeekseweg 60, 5022 GC Tilburg, The Netherlands

**Keywords:** Radiology podcast, Social media, Podcasts

## Abstract

**Objectives:**

Podcasts are audio recordings distributed via the Internet. We review the availability of podcasts on the topic of radiology.

**Methods:**

A search for podcasts relating to radiology was performed using search engines and free public websites that either hosted or distributed podcasts. Only English language podcast series were included, and video podcasts were excluded. Data was gathered by manually interrogating the metadata on the primary hosting platform and related websites.

**Results:**

Forty-one podcast series met the inclusion criteria. The earliest was from 2005. In total, 56.1% of podcasts were defined as active and 43.9% inactive at the time of publication. Number of episodes for each podcast series ranged from 1 to 269 with 56.1% of podcasts having ≤ 10 episodes. There was a wide variation in podcast series’ frequency/schedules. The most common subject topic was ‘radiology current affairs’ (43.9%), with the least common ‘exam revision’ (7.3%) and ‘radiography’ (7.3%). The majority of podcasts were targeted at radiologists (87.8%) and originated from the USA (70.1%). Podcast hosts consisted of doctors (63.4%), other professionals (29.3%) or unknown (7.3%). Additional supplementary media or information as show notes were provided by 26.8% of radiology podcast series.

**Conclusions:**

This gives a new insight into the world of ‘radiology podcasting’. To the authors’ knowledge, this is the first review in the literature and highlights the increasing availability of podcasting in radiology.

## Key points

- Podcasts provide a novel means for radiology focused educational content.
- There are a varied and growing number of radiology podcasts.

## Introduction

Podcasts are audio recordings distributed via the Internet and available to download or stream on smartphones, tablets, portable devices and personal computers without payment. A podcast episode is a single audio recording, and multiple episodes under the same umbrella title make up a podcast series. Users typically subscribe to podcasts via podcast apps to allow automatic downloading of new episodes. They are convenient as they may be listened to on almost any Internet-enabled device such as smartphones, computers and smart speakers, at any time, for example, whilst doing other activities such as driving [1].

There has been rapid growth in the awareness of podcasting with an estimated 70% of the US population now familiar with this term [2]. An estimated 32% of Americans (age 12+) listen to a podcast every month in 2019, compared with 9% in 2008 [2]. This percentage was higher in younger listeners with an estimated 40% of those aged 12–24 and 39% of those aged 25–54 listening to at least one podcast each month [2]. Much of this growth is believed to be driven by smartphone use [3]. In 2019, 93% of podcast listeners listened to either ‘most of’ or the entire podcast episode [1].

Some podcasts are published along with ‘show notes’ which may include a written transcript of the audio, links to resources and other supplementary information. Podcasts require minimal technical knowledge to create and may be of high quality using a microphone and audio editing software, but may also be recorded and created using a smartphone [4].

The aim of this article is to review the availability of podcasts on the topic of radiology.

## Material and method

### Identification of podcast series

All information was sourced from free public websites that either hosted or distributed podcasts.

To ensure a comprehensive search of available free podcasts, a search was performed using the search term ‘radiology’ on 10 podcast hosting platforms: Anchor, Apple Podcasts/iTunes, Spotify, Google Podcasts, Podbean, Overcast, Stitcher, Breaker, Castbox and RadioPublic (Table 1).

**Table 1:** Web addresses for each hosting platform searched

Hosting platform	Web address (URL)
Anchor	https://anchor.fm
Apple Podcasts/iTunes	https://www.apple.com/uk/itunes/podcasts
Spotify	https://www.spotify.com
Google Podcasts	https://podcasts.google.com
Podbean	https://www.podbean.com
Overcast	https://overcast.fm
Stitcher	https://www.stitcher.com
Breaker	https://www.breaker.audio
Castbox	https://castbox.fm
RadioPublic	https://radiopublic.com

An additional search in Google using the search term ‘radiology podcast’ was performed, and the first 10 result pages were explored to look for any additional podcasts not identified by our initial search and ensure as comprehensive a search as possible. It is not possible to guarantee that every eligible podcast series has been included in this study as podcasts are a decentralised medium and there is no single exhaustive database. However, we hope that by looking at multiple different hosting platforms and sources, that our search is more extensive than similar studies which have used the ‘iTunes podcast directory’ as the primary source for data collection [5].

### Inclusion and exclusion criteria

To ensure only valid podcast series were included, stringent inclusion and exclusion criteria were set as follows:

- The overarching theme of the podcast series must be radiology. Podcast series with episodes related to radiology, but where radiology was not the primary theme of the whole series were excluded.
- Podcasts hosted solely on paid-for subscription platforms were excluded.
- Video podcasts, also called ‘vodcasts’, were excluded. A vodcast is defined as a podcast that contains video content. If a video podcast had a separate pure audio offering, then this was included.
- Only English language podcast series were included.

### Data collection and categorisation

On 12 October 2019, the authors collected various information relating to each podcast series as summarised in Table 2. Data was gathered by manually interrogating the visual and textual ‘metadata’ relating to each podcast series on the primary hosting platform and related websites, e.g. social media pages. Where necessary, the audio content of the podcast series was also listened to by the authors to obtain required information that was not included in this visual and textual metadata such as the name of a particular podcast host. It was not necessary to contact any individuals directly associated with the podcasts to obtain any of the data required. All data was manually coded and categorised by the authors. The associated supplementary database contains specific dates of when information pertaining to each podcast was gathered.

**Table 2:** Information collected for each podcast series along with description/definition

Information	Description/definition
Podcast title	Title given by the podcast authors on website or hosting site.
Subject category	We devised four categories into which we classified the various podcast series based on common subject matter. These were as follows: • Journal podcast—podcasts affiliated with a peer reviewed journal or in which the review of journal articles was the subject matter. • Exam revision—podcasts intended specifically to be used as a revision resource for a named radiology exam. • Educational—podcasts aimed at educating listeners, but not directly related to exam revision or affiliated with a peer reviewed journal. • Radiology current affairs—podcasts that discuss issues relevant to clinical radiology. • Radiography—podcasts intended for radiographers (radiologic technologists).
Network/affiliation	Organisation, institution and/or body affiliated with the podcast series.
Hosts	The host/interviewer for the podcast series was categorised as one of the following: • Named host(s) • Anonymous (host not named) • Various (no regular host, e.g. if the podcast series consisted of presentations rather than interview/discussion format)
Number of hosts	Number of hosts/interviewers per podcast series (guests not included). If the number of interviewers/speakers varies, then ‘variable’ is stated.
Country	Name of the country primarily associated with the podcast.
Show notes	Supplementary media or information on the podcast episodes available to audiences via podcast apps or related websites (if present). Show notes may include images, videos, hyperlinks, scientific references and audio transcripts; however, simple descriptions of a podcast episode are not classified as ‘show notes’.
First episode	Date the first published episode was available to stream or download.
Most recent episode	Date the most recent published episode was available to stream or download.
Lifetime	Duration between the first episode becoming available to stream or download, and the most recent episode.
Sampling date	Date on which data was collected.
Number of episodes	Total number of episodes available to stream or download in the podcast series to date.
Frequency schedule	Regularity with which podcast episodes are released. If there was a slight variation between podcast releases, then an average schedule was determined at the discretion of the author: • Weekly—once a week • Fortnightly—two episodes every month • Monthly—once a month • Quarterly—once every 3 months • Sporadic—no pattern of regularity • Undetermined—insufficient episodes to determine frequency
Status	Whether podcast is active or inactive. Definitions are as follows: • Active—most recent episode released within 6 months (180 days) of sampling date. • Inactive—no episodes released for 6 months (180 days) prior to the sampling date.
Website	Address of website or dedicated section/page on the network/organisation website dedicated to the podcast (if present).
Twitter	Twitter account or network/organisation Twitter account which promotes the podcast content (if present).
Facebook	Facebook account or network/organisation Facebook account which promotes the podcast content (if present).
Email	Is a contact email address provided for the podcast?

### Statistical analysis

All data relating to the podcast series and subsequent categorisation was recorded in a spreadsheet (Google® Sheets), and basic descriptive and categorical analysis was undertaken within the spreadsheet. The figures were created using Google Sheets and R 3.6.1 (R Core Team (2019). R: A language and environment for statistical computing. R Foundation for Statistical Computing, Vienna, Austria. URL https://www.R-project.org/).

## Results

**Fig. 1:**
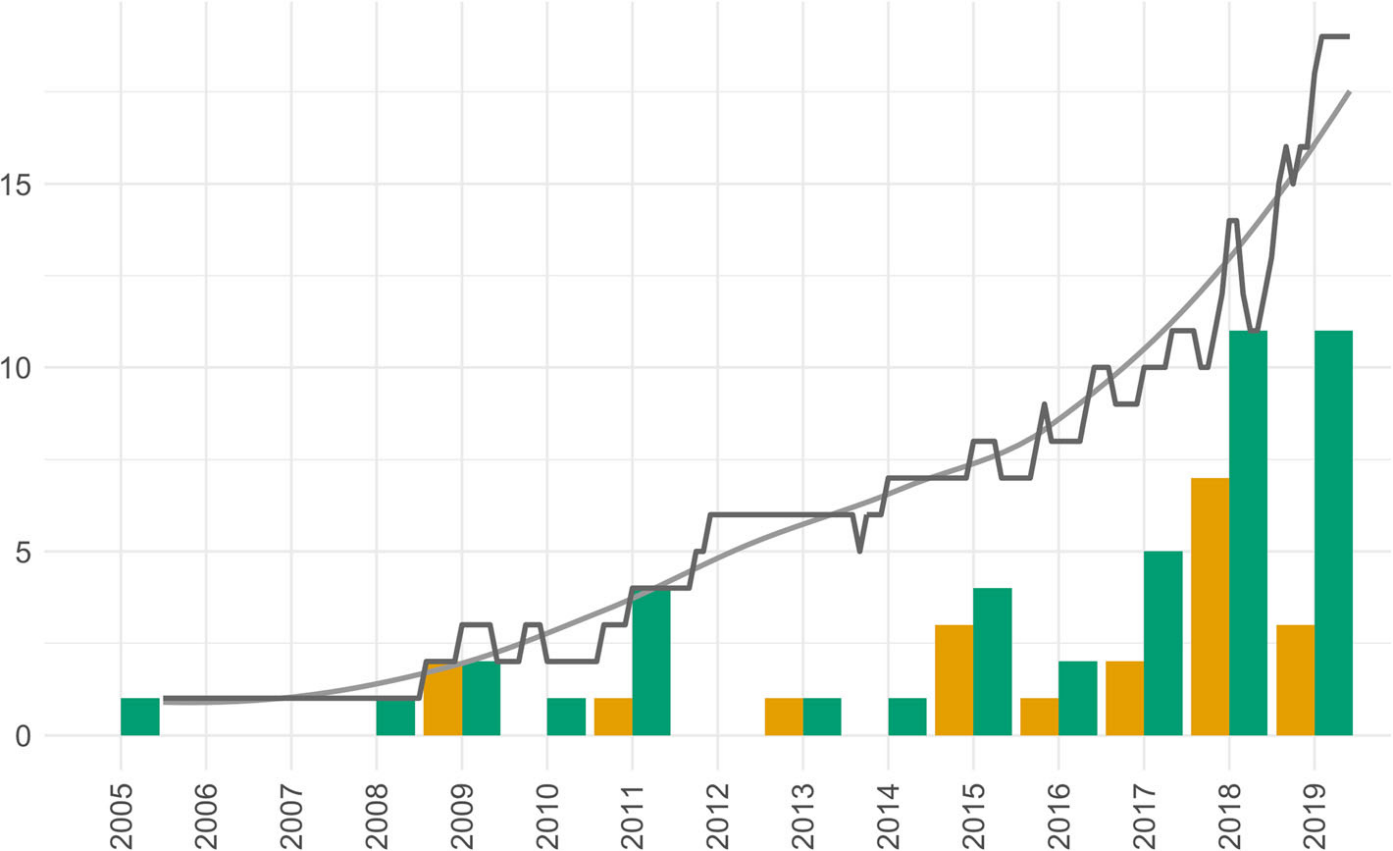
Development of podcasts over time. The black line shows the total number of active podcast series (*y*-axis) by year (*x*-axis). The bar chart shows the number of podcast series started (green) and discontinued (orange) in each year. The graph shows that the total number of podcasts has risen steadily over the last 14 years, with a more rapid increase in the number of new podcasts over the last 2 years

Forty-one podcast series met the inclusion criteria for the study (Table 3). The earliest radiology podcast started in 2005, and at the time of writing, there were 10 new radiology podcasts in 2019. Twenty-three of the 41 podcast series (56.1%) were defined as active, meaning that they had released an episode within the immediate 6 months prior to our search. Eighteen of the 41 podcast series (43.9%) were inactive. Some podcast series had been inactive for longer than others (range 195–3811 days). Overall, there was a steady increase in the total number of podcasts and active podcasts over time (Fig. 1). Interestingly, there were a large number of new podcasts within the last 12 months (*n* = 12).

**Table 3:** Summary of radiology podcast series (data sampled 12 October 2019)

Podcast title	Category	Affiliation	Country	Number of episodes	Active/inactive
5 Minute Radiography	Radiography	None	USA	8	Inactive
A Look Inside	Educational	None	USA	2	Inactive
Advances in Medical Imaging	Radiology current affairs	Reach MD	USA	22	Inactive
Advancing Clinical Research Quibim Podcast	Radiology current affairs	Quibim	USA	9	Inactive
AJNR Podcasts	Journal podcast	American Journal of Neuroradiology	USA	227	Active
British Institute of Radiology Podcasts	Radiology current affairs	British Institute of Radiology	UK	58	Active
Capitol Radiology	Educational	Capital Radiology	Australia	2	Inactive
Cassling Academy	Radiology current affairs	None	USA	5	Active
Clinical Correlation Required	Educational	None	USA	3	Inactive
Clinical PET Cast	Educational	None	USA	50	Inactive
Clinical Radiology	Journal podcast	Royal College of Radiology	UK	30	Active
CTisus	Radiology current affairs	None	USA	66	Inactive
Diagnostic Imaging	Radiology current affairs	Modern Medicine Network	USA	21	Inactive
Diffusion - A quick radiology podcast by Bhavin Jhankaria	Educational	None	India	1	Active
EuSoMII on AIR	Radiology current affairs	European Society of Medical Imaging Informatics	Austria	4	Active
Exploring the Horizon	Radiology current affairs	None	Belgium	9	Active
JVIR	Journal podcast	Journal of Vascular and Interventional Radiology	USA	89	Active
Meaningful Use In Radiology	Radiology current affairs	Carestream	USA	5	Inactive
Medical Imaging Matters	Radiology current affairs	The Association for Medical Imaging Management	USA	2	Active
Medscape Radiology Podcast	Radiology current affairs	Medscape	USA	45	Inactive
Men In Lead Aprons	Educational	Center for Radiological Research	USA	10	Inactive
More Programs in Radiology	Radiology current affairs	University of California Television	USA	24	Active
Philips Healthcare Talks	Radiology current affairs	Philips	USA	6	Inactive
Philips Imaging Connections	Radiology current affairs	Philips	USA	3	Active
Pro Tips on How to Write Outstanding Radiology Letters of Recommendation	Educational	Writeperfect LTD	Cyprus	1	Active
R25	Journal podcast	NYU Langone Medical Center in New York City	USA	6	Inactive
RadCast	Radiology current affairs	None	UK	18	Active
Radiographics Podcasts	Journal podcast	Radiological Society of North America	USA	39	Active
Radiology Firing Line	Journal podcast	Journal of the American College of Radiology	USA	61	Active
Radiology Podcasts	Journal podcast	Radiological Society of North America	USA	269	Active
RadProfPodcast	Educational	None	USA	1	Inactive
Rapids by Songs 4 FRCR	Exam revision	None	UK	1	Inactive
RLI Taking the Lead Podcast	Radiology current affairs	American College of Radiology	USA	34	Active
Rural Radiology Journal club	Journal podcast	Australia and New Zealand Rural Radiology Special Interest Group	Australia and New Zealand	2	Inactive
SIIMcast	Radiology current affairs	Society for Imaging Informatics in Medicine	USA	33	Active
Songs 4 FRCR: Radiology FRCR 2A Revision	Exam Revision	None	UK	25	Active
The FRCR 2B Podcast	Exam revision	Imperial College Radiology Training Scheme	UK	6	Active
The Hounsfield Unit	Educational	None	USA	2	Active
The Radiologic Technologist Podcast	Radiography	None	USA	2	Active
The Sound of IR Podcast	Radiology current affairs	None	USA	26	Active
The Topics in Radiography Podcast	Radiography	None	USA	9	Inactive

**Fig. 2:**
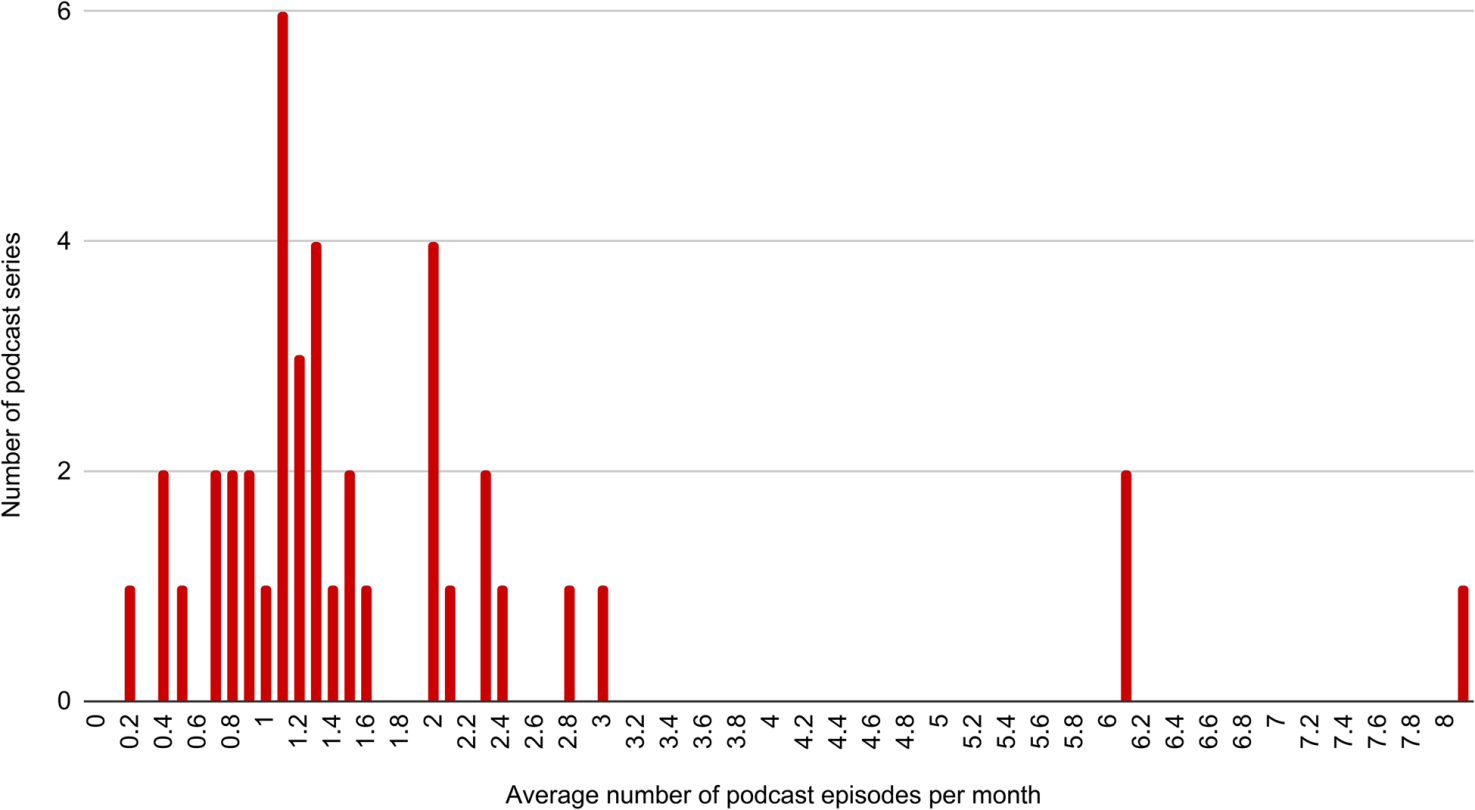
The average number of episodes per month over the lifetime of each podcast. The graph shows the average rate at which episodes are released by each podcast series. The graph shows that most podcasts released new episodes on average once a month or more frequently

The number of episodes released by each podcast series was highly variable ranging from 1 to 269 episodes. The average number of episodes per month over the lifetime of each podcast series (time from the first podcast episode to the most recent podcast episode) ranged from 0.2 to 8 (Fig. 2). 56.1% of podcasts had 10 episodes or fewer, and 17% had 50 episodes or more. The mean number of episodes was 30.1, and the median was 9.

**Fig. 3:**
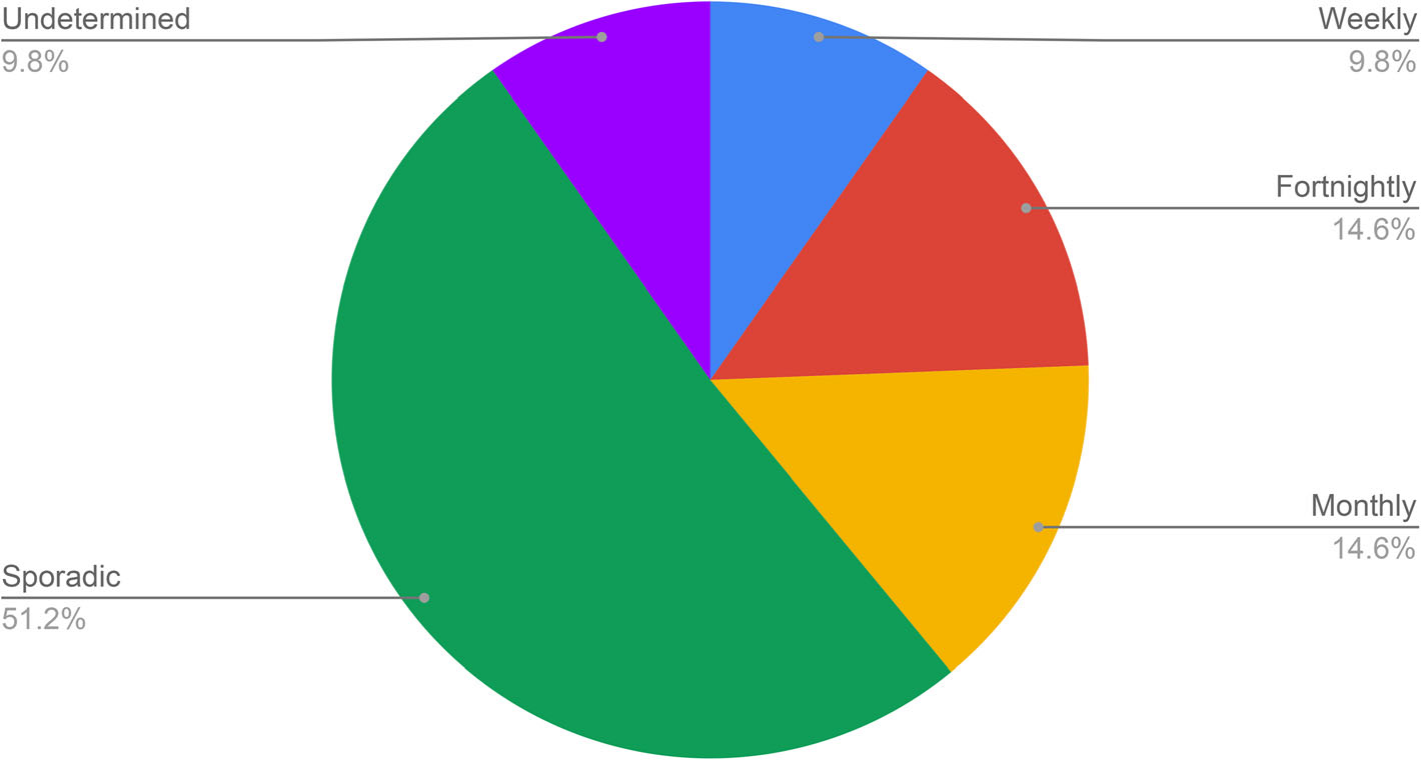
Podcast series’ frequency/schedule. The pie chart shows the distribution of scheduling of new podcast episodes for each podcast series. The largest proportion sampled were sporadic (51.2%), followed by monthly and fortnightly (both 14.6%), then weekly and undetermined (both 9.8%)

There was a wide variation in podcast series’ frequency/schedules (Fig. 3), with most podcasts published on a sporadic basis; 4 were weekly, 6 fortnightly, 6 monthly, 0 quarterly, 21 sporadic and 4 undetermined as there were insufficient episodes to determine frequency.

**Fig. 4:**
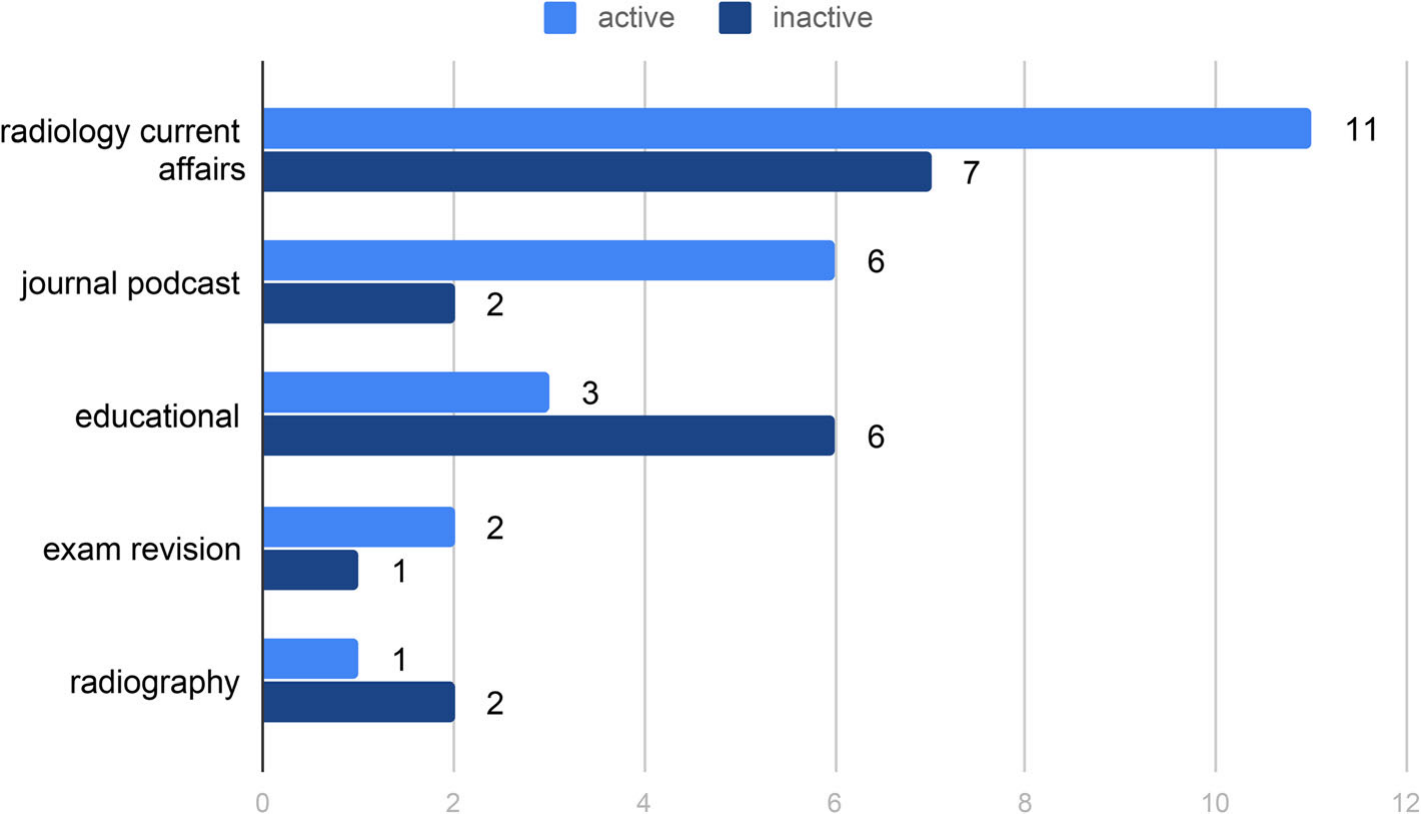
Podcast series by category and whether active or inactive. A bar chart showing the number of podcast series in each subject category and whether active or inactive.

When grouped by subject category, most podcast series were on the topic of ‘radiology current affairs’ (43.9%), followed by ‘educational’ (22%), ‘journal podcast’ (19.5%), ‘exam revision’ (7.3%) and ‘radiography’ (7.3%). Figure 4 shows the number of podcast series in each category and whether active or inactive.

The vast majority of radiology podcasts were targeted at radiologists (87.8%) with three targeted at radiographers and students (7.3%), one at data scientists (2.4%) and one at healthcare companies (2.4%).

When we looked at the country primarily associated with each podcast series, the majority originated from the USA (70.1%), followed by the UK (14.6%) then Australia/New Zealand (4.9%) followed by Austria (2.4%), Belgium (2.4%), Cyprus (2.4%) and India (2.4%).

Additional supplementary media or information as show notes were provided by 26.8% of radiology podcast series radiology podcast series in the form of article links (17.1%), podcast transcripts (9.8%), revision notes (4.9%) and videos (2.4%). Two podcasts provided both article links and podcast transcripts (those published by the Radiological Society of North America).

36.6% of podcasts were hosted by radiologists, 12.2% by radiologists in training (junior doctors/residents), 12.2% by doctors outside of radiology, 12.2% by radiographers, 2.4% by a medical student, and 17.1% by other professionals (content development manager, senior counsellor, academic, corporate director/president), and in 7.3%, the host regularly changed and it was unknown who the host was. 85.4% had a single interviewer/host, and 14.6% had more than one host/interviewer.

## Discussion

To the authors’ knowledge, this is the first time radiology podcasts have been analysed in the literature and this gives a new insight into the world of ‘radiology podcasting’. The authors searched the PubMed database, and no similar review papers could be found.

Podcasting usage has shown a steady increase over time [2]. There are now over 700,000 active podcasts with over 29 million podcast episodes [6]. As can be seen above, a significant number of radiology-related podcasts are already available on a range of platforms. There is currently no detailed data available on the age or demographics of the audience reached by those radiology-related podcasts; however, data from the general podcasting suggest that the vast majority of listeners should fall in the 25–44 years of age group and be almost equally distributed with respect to gender [6]. With that in mind, it would only seem logical that most radiology-related podcasts currently focus on content that is relevant to this target group and features either specific educational material or topics that may be of special interest to an audience at the beginning or in the early middle of their career.

Outside of radiology, among the genres most listened to, educational podcasts are currently the second most listened to genre, surpassed only by comedy podcasts [7]. Therefore, it is interesting to note that of the nine educational podcasts in our study, only three remain active. We cannot be sure of the reason for this, but there are many factors to consider such as ‘educational’ in the general setting may include journal podcasts and other scientific podcasts, which we categorised separately in our paper. The high proportion of inactive podcasts is reflective of a wider phenomenon across all podcast genres known as ‘podfade’. This is when a podcast ceases to publish new episodes [8]. In a 2015 semi-formal non-peer-reviewed study looking at all podcasts on iTunes US, Morgan found that only 40% of podcasts were active (had released an episode in the last 6 months) [9]. In 2019, MacKenzie, looking specifically at science podcasts, found only 46% to be active (had released an episode in the last 3 months) [5]. Our study found that overall 56.1% of radiology podcasts were active which is in line with the literature and indicates the difficulty of maintaining a podcast series. Reasons for podfade include loss of passion, lack of time, loss of a co-host and major life events [10]. We also hypothesise that the hosts of the educational podcasts may have stopped producing episodes because they progressed in their careers and the original aim of the podcast (such as for exam revision, for example) was no longer relevant to them, or that maybe they lacked funding for the podcast since usually there is no reimbursement unless sponsored by a company or a society.

Although some could argue that educational activities requiring dedicated attention such as reading a book or listening to a live lecture could have a more lasting effect, some studies suggest that the retention rate is relatively high from purely auditory learning [7]. It also seems that podcast listeners listen to podcasts in a way that allows for multitasking whilst listening. This is reflected by the results of a recent survey which suggested that a significant percentage of users listen to podcasts whilst driving (52%), commuting on public transport (37%), travelling (46%) or even during workout (32%) [7]. A recent qualitative study on a medical podcast found that the podcast was perceived to increase efficiency and permit users to multitask by using mobile platforms [11]. Interestingly, surveys also found that almost a fifth of all podcast listeners increased the speed of the podcast to be able to listen to it faster [6].

The authors feel that the ‘radiology podcasts’ from RSNA deserves a special mention since they have produced a continuous programme of podcasts for over 10 years and the highest number of radiology podcasts so far with 269 episodes. The host is David Bluemke (editor of *Radiology*), and the authors felt the quality was high with topics well-selected, which could be explained by the fact that the content is related to the content of the journal, which is one of the most highly ranked radiology journals [12]. As well as interesting summaries of latest journal articles, they also interview authors and we feel this is an excellent example of what a radiology podcast can be.

Despite not meeting our inclusion criteria, we feel the podcast series ‘Professor Hallux’ also deserves an honourable mention due to its novelty. This is a podcast produced by the educational and entertainment website FunKidsLive.com, designed to educate children on the human body. As part of a collaboration with the Royal College of Radiologists (UK), they produced a ten-part series called ‘Looking Inside the Human Body’ to introduce children to radiology. Episodes included ‘Who’s who in radiology’, ‘How ultrasound works’ and ‘The science behind radiation’. As this was only a short run of episodes within a much larger series unrelated to radiology, it was judged not to meet our inclusion criteria and excluded from our results.

Although the authors tried their best to obtain high-quality data, it is important to note the limitations of this study. Firstly, there is no single directory within which all podcasts can be found. Therefore, although we tried to be as comprehensive as possible, it is still possible that there are eligible podcasts that have not been included in our study. Given the extent of our search, however, it is unlikely that an eligible podcast not included in our study would be found by a member of the target audience. Secondly, only podcasts in the English language were assessed, so there may be radiology podcasts in other languages which we have not included. Thirdly, podcast episode length and statistics on the number of downloads were not publically available. This data would have been useful to gain a more complete analysis of the use and proliferation of radiology podcasts. We did not formally evaluate the quality of individual podcasts or analyse the motivations for podcast hosts to create these podcasts, and other than the presence of show notes, we did not look into how the various podcasts used social media or other websites to engage with audiences. In several cases, the identity of the podcast presenter(s) and their credentials were not openly declared, and so this was difficult to ascertain. This poses a problem as listeners may be receiving information from non-credible or unverifiable sources. In such cases, we were also unable to analyse the authors motivation for hosting the podcast and scrutinise for conflicting interests.

To conclude, this study is the first to look at the phenomenon of ‘radiology podcasts’ and shows that there are an increasing number of radiology podcasts with a large number of new podcasts in the last 12 months, with the majority targeted at radiologists. The reasons behind this increase were not assessed; however, it fits with the trend of growing use of podcasting in general. Further research regarding the profile of the podcast audience, the listener motivation and experience, and the most appreciated type of content for radiology podcasts would be valuable for optimising usage of this medium.

## Data Availability

All data is publicly available and can be obtained by following the steps as specified in the ‘Material and method’ section.
